# Maternal death reviews at Bugando hospital north-western Tanzania: a 2008–2012 retrospective analysis

**DOI:** 10.1186/s12884-015-0781-z

**Published:** 2015-12-15

**Authors:** Moke Magoma, Antony Massinde, Charles Majinge, Richard Rumanyika, Albert Kihunrwa, Balthazar Gomodoka

**Affiliations:** Evidence for Action Project Tanzania, P.O.BOX 1371, Dar es salaam, Tanzania; Department of Obstetrics & Gynaecology, Bugando Medical Centre, Mwanza, Tanzania

**Keywords:** Maternal death reviews, Hospital, Tanzania

## Abstract

**Background:**

Unacceptably high levels of maternal deaths still occur in many sub-Saharan countries and the health systems may not favour effective use of lessons from maternal death reviews to improve maternal survival. We report results from the analysis of data from maternal death reviews at Bugando Medical Centre north-western Tanzania in the period 2008–2012 and highlight the process, challenges and how the analysis provided a better understanding of maternal deaths.

**Methodology:**

Retrospective analysis using maternal death review data and extraction of missing information from patients’ files. Analysis was done in STATA statistical package into frequencies and means ± SD and median with 95 % CI for categorical and numerical data respectively.

**Results:**

There were 80 deaths; mean age of the deceased 27.1 ± 6.2 years and a median hospital stay of 11.0 days [95 % CI 11.0–15.3]. Most deaths were from direct obstetric causes (90); 60 % from eclampsia, severe pre-eclampsia, sepsis, abortion and anaesthetic complications. Information on ANC attendance was recorded in 36.2 % of the forms and gestation age of the pregnancy resulting into the death in 23.8 %. Sixty one deaths (76.3 %) occurred after delivery. The mode of delivery, place of delivery and delivery assistant were recorded in 44 (72.1), 38 (62.3) and 23 (37.7 %) respectively.

**Conclusion:**

Routine maternal death reviews in this setting do not involve comprehensive documentation of all relevant information, including actions taken to address some identified systemic weaknesses. Periodic analysis of available data may allow better understanding of vital information to improve the quality of maternity care.

## Background

Maternal mortality remains high in most sub-Saharan African countries, including Tanzania.[1, 2] Even among women who seek care in health facilities for pregnancy-related complications early enough, case fatality rates may be high [3], indicating sub-optimal care and the imperative of improving the quality of care for better maternal survival.

The maternal mortality ratio in Tanzania is 454 deaths per 100,000 [4] and levels have not declined significantly in the past two decades to meet the 5^th^ millennium development goal by 2015. [2] Direct obstetric causes of maternal deaths account for at least three quarters of these deaths [1, 3], and although life-threatening pregnancy-related complications may start at home for some women, most come into contact with the health system before they die [3]. Sub-optimal care is also common in health facilities. [5] Utilization of essential services for maternal and newborn survival such as skilled delivery attendance and emergency obstetric care is not always possible even among women living within 5 km of health facilities. [3, 6].

Of the at least 7900 maternal and 39,000 newborn deaths expected to occur in Tanzania in 2013 [2, 7], a significant number could have been saved with better quality of care in health facilities as critical skills, equipment, drugs and other essential commodities for saving maternal and newborn deaths often lack. [8–10] Nevertheless, lessons learnt from managing individual cases may be equally important for improving the quality of care women receive in health facilities. For example, gaps identified in a near miss or maternal deaths reviews can inform providers on how to avoid similar future deaths. One strategy advocated to address the latter is health facility maternal death review [11, 12]-defined as a qualitative, in-depth investigation of the causes of and circumstances surrounding maternal deaths occurring at health facilities [11].

In Tanzania, guidelines for maternal and perinatal death reviews (MPDRs) were produced and rolled out in 2006. The guidelines aim to improve the process of maternal death reviews with an ultimate goal of improving case management for better maternal and newborn survival. [13] They also require a review team in each health facility (membership eligibility stipulated) which is supposed to meet within 24 h of the death occurring. Typically, a review session for a maternal death at a health facility takes approximately one to two hours and the process involves presentation of a case summary by someone who was not involved in the case management, identification of gaps and strengths in case management and drafting of an action plan with timelines and specific roles to the identified levels of care to act on the recommendation (s) in order to prevent similar deaths in future. Review committees are also required at all other levels of health administration in the country (district, region and national) to meet at stipulated times. But available evidence suggests that except for health facility and district meetings, regional level meeting are not always convened and no national meeting has ever been convened. [14] Review reports from health facilities are subsequently forwarded to the district where reports are also sent to the region and to the responsible section within the Ministry of Health and Social Welfare.

Whilst most deaths in health facilities are possibly reviewed and reported, implementation of these guidelines has not yielded much evidence to support that the maternal death reviews (MDRs), although conducted routinely, provide results that are acted upon to improve the quality of maternal and newborn health services. Maternal and perinatal death forms are filled routinely and sent to various levels as stipulated in the guidelines, but evidence of critical analysis of whether results from such reviews are communicated back, or even facility level recommendations are acted upon is scarce. [14] Furthermore, emerging evidence from epidemiological studies in the country suggests that despite significant improvement in utilization of health facilities among women with life-threatening pregnancy-related complication in recent years [3], maternal deaths have not decreased proportionately [1, 2, 4]; suggesting sub-optimal quality of care. MDRs aim to provide lessons to prevent similar subsequent maternal death events in health facilities, but if women continue to die in health facilities for causes that are identified routinely, doubts are abound for the effectiveness of the current MDR strategy to address the high level of such deaths. Furthermore, the country is currently reviewing the MPDR guidelines to align with the global demand for surveillance and response as the better way of understanding and responding to the levels, causes and trends of maternal mortality. [15–17].

We report the results from the analysis of data from maternal death reviews at Bugando Medical Centre 2008–2012, a tertiary hospital in Mwanza Tanzania and discuss how lessons from the results have helped to institute measures to improve care provided to women with maternal complications in the institution. The results from the analysis were also used to inform an on-going review of the existing MPDR guidelines in the country.

## Methodology

The study is a retrospective analysis of maternal death reviews at a tertiary hospital north-western Tanzania in the period 2008–2012. This is a 900 bed capacity and the main referral hospital for the population in the Lake and western zones constituting almost 37 % of the entire Tanzania mainland population, estimated at 43,625,354 people in 2012. [18] There are about 7000 deliveries at this hospital annually, most being referrals from lower level health facilities.

All copies of the reviewed maternal deaths covering the study period were accessed (80 deaths). Typically, maternal deaths reviews are conducted as they occur, usually within a week of occurring by a team led by senior obstetricians in a morning meeting. Case summaries of deaths are prepared by resident doctors in obstetrics and gynaecology not involved in the case management (usually one such doctor is assigned a case to summarize and present). In contrast to what is stipulated in the national guidelines on who should be involved in the reviews, the circumstance at the hospital demands teaching of both undergraduate medical and nursing students who are invited in such meeting as part of learning the process. Senior midwives in the department are also involved in the review besides clinicians and midwives involved in the case management. However, anaesthetists and pharmacists are rarely invited to participate.

Once the review has been done, copies of the review findings are sent to the hospital Director and the zonal reproductive and child health office housed at the hospital. The latter is responsible for forwarding the filled forms to various levels of reporting; usually to the district reproductive and child health coordinator who also forwards the information to the regional and national offices quarterly.

We reviewed multiple data sources at the hospital for deaths that occurred in this period: labour ward, medical records, theatre registers and emergency and casualty department. Missing information for two deaths not included in the existing maternal deaths review forms were identified. Corresponding review forms were found in the deceased patients’ files and included in the analysis.

Information on the forms were entered into an excel spreadsheet and missing information was extracted from case notes in patients’ files if available. Data were then transferred into Stata statistical package in windows version 12 (StataCorp LP College Station, Texas, USA) for cleaning, coding and analysis. Categorical data were analysed into frequencies to understand the various components of review that were completed and filled and causes of deaths and associated factors while numerical data into means ± SD, range as well as median with 95 % CI.

The analysis is part of a larger study titled “Improving quality of maternal and newborn care in Tanzanian health facilities: Lessons from a mixed method assessment of health facility maternal deaths reviews in the Lake zone of north-western Tanzania” with research permits from the Catholic University of Health and Allied Sciences (CUHAS)/Bugando Medical Centre and National Institute for Medical Research (NIMR) ethical committees (no CREC/018/2013 & NIMR/HQ/R.8a/Vol.IX/1543 respectively). Permission for the retrospective access to the deceased patients’ hospital records was also requested and obtained from the administration of Bugando Medical Centre, including the head of the department of obstetrics and gynaecology as required by the national regulations. Data extraction was done by one of the authors who was not involved in the management of any of the patients and de-linked the deceased patients’ information to ensure data anonymity in the subsequent steps of data handling.

## Results

Table 1 shows some characteristics of the maternal deaths. Most were young with the mean age of 27.1 ± 6.2 years (range 15–41 years). The majority were aged 20–35 years (80 %), had unknown level of education (57.5 %) and resided in Mwanza region (83.5 %). The median age was 26 years (95 % CI 25–29) (information not shown in the table). Almost 74 % were housewives or peasant farmers and married (88.8 %).

**Table 1:** Characteristics of maternal deaths at Bugando Medical Centre 2008–2012

Characteristic	Number	%of total	Mean ± SD
Age of the diseased in years *n* = 80			
Overall			27.1 ± 6.2
<20	8	10.0	
20–35	64	80.0	
>35	8	10.0	
Total	80	100.0	
Regional of residence of the diseased *n* = 79			
Mwanza	66	83.5	
Mara	7	8.9	
Shinyanga	5	6.3	
Tabora	1	1.3	
Total	79	100.0	
Level of education of the diseased *n* = 80			
None	2	2.5	
Primary education	26	32.5	
Secondary and above	3	3.8	
Above secondary	3	3.8	
Unknown	46	57.5	
Total	80	100.0	
Recorded occupation of the diseased *n* = 69			
Housewife	29	42.0	
Peasant farmer	22	31.9	
Teacher	3	4.4	
Student	3	4.4	
Businesswomen	2	2.9	
Petty trader	1	1.5	
Housemaid	1	1.5	
Tailor	1	1.5	
Police	1	1.5	
Unknown	6	8.7	
Total	69	100.0	
Religion of the diseased *n* = 67			
Christian	47	70.2	
Muslim	18	26.9	
Others	2	2.9	
Total	67	100.0	
Marital status of the diseased *n* = 80			
Divorced	1	1.3	
Cohabiting	2	2.5	
Single	6	7.5	
Married	71	88.8	
Total	80	100.0	

Table 2 shows additional characteristics of the deceased women. Few were five times or more pregnant (11.3 %). On average, a patient stayed at Bugando hospital for almost two weeks before death (mean 13.5 ± 5.7 days) and median 11.0 [95 % CI 11.0–15.3] (not shown in the table) and just 20 % stayed for less than one day.

**Table 2:** Additional characteristics of maternal deaths at Bugando Medical Centre 2008–2012

Number of children ever delivered by the diseased	Number	% of total	Mean ± SD
Overall			
Never delivered before	9	11.3	
≤4 children	62	77.4	
≥5	9	11.3	
Total	80	100.0	
Duration of stay at Bugando Hospital (in days)			
Overall			13.5 ± 5.7
<1	16	20.0	
1–3	38	47.5	
≥4	26	32.5	
Total	80	100.0	
Some important information recorded during death review			
Information on whether attended ANC *n* = 80	29	36.2	
Information on gestation age of the pregnancy resulting into the death *n* = 80	19	23.8	
Place of delivery documented (those recorded to had delivered) *n* = 61	38	62.3	
Gestation age at delivery recorded *n* = 61	23	37.7	
Delivery assistant recorded *n* = 61	29	47.5	
Mode of delivery recorded (those recorded to had delivered) *n* = 61	44	72.1	
Highlighted direct causes of deaths	76	95.0	
Eclampsia	16	20.0	
Sepsis	14	17.5	
Postpartum haemorrhage	9	11.3	
Abortion	8	10.0	
Anaesthetic complications	8	10.0	
Severe pre-eclampsia	5	6.2	
Embolism	3	3.7	
Mentioned as direct but no specific causes highlighted	13	16.3	
Highlighted indirect causes of deaths	4	5.0	
AIDS related	3	3.8	
Heart Diseases	1	1.2	
Total	80	100.0	
Identified factors associated with maternal deaths			
Late referral from another facility	21	30.9	
Delay in receiving appropriate treatment	15	22.1	
Delay in deciding to seek care	14	20.6	
Inadequate provider skills	8	11.8	
Lack of supplies and equipment in health facilities	6	8.8	
Inadequate staff in health facilities	3	4.4	
Poor infrastructure for quick referral	1	1.4	
Total	68	100.0	

Note: Deaths from obstructed labour and anaemia were categorized as contributing factors and not causes and classified as of sepsis or haemorrhage

In most cases, the review forms were incompletely filled. Of the total 80 reviewed deaths, only 29 (36.2 %) had information on whether the woman attended ANC and 19 (23.8 %) had information on gestation age of the pregnancy resulting into the death. Sixty one deaths (76.3 %) occurred after the women had delivered and of these the mode of delivery was recorded in 44 (72.1), 38 (62.3 %) had place of delivery recorded, 29 (47.5 %) had the delivery assistant recorded and only 23 (37.7 %) had gestation age at delivery recorded. In addition, only 39 (62.9 %) had the outcome of delivery recorded; 25 (41 %) with live birth, 11 (18.0) fresh stillbirth and the remaining three (4.9 %) macerated (information not shown in the tables).

Half of the total deaths (40 out of 80) had information on whether a post-mortem was performed or not. Likewise, filling in the causes of death as required by the forms (both direct and indirect causes) were not always done. Of the total 80 deaths, information was filled for only 72 (90); 58 (72.5 %) with direct causes highlighted but 16.3 % of the deaths although identified as such, specific causes were not filled in the review forms. Eclampsia and severe pre-eclampsia accounted for 26.2 % of all deaths while sepsis and postpartum haemorrhage accounted for 17.5 and 11.3 % of all deaths respectively. Both abortion and anaesthetic complications accounted for one in ten of all deaths. The filled indirect causes of deaths did not provide an in-depth understanding of the individual causes but 5 % of the deaths were attributed to indirect causes with AIDS accounting for 3.8 % of all deaths. Of the total 80 deaths, 68 (85 %) had various factors associated with the deaths identified and recorded on the review forms. Late referral from another health facility, delay in receiving appropriate care at the referring health facility and at Bugando Medical Centre and delay in deciding to seek care were the commonest factors identified.

Furthermore, complete data to allow analysis of all deaths on whether they occurred before, during or after delivery as well as on actions that were suggested during the review meetings and possibly taken thereafter were missing, an indication of the quality of the meetings.

## Discussion

The study highlights some issues with maternal deaths review at a tertiary health facility in Tanzania. Notably, maternal death reviews are conducted but relevant information is not always recorded as per available guidelines. The missing information includes planned actions to prevent similar deaths in future, thus defeating the purpose of the reviews. Nevertheless, the retrospective analysis allowed for identification of such bottlenecks and institution of appropriate measures such as review of the management for eclampsia and severe pre-eclampsia patients to include admission to a high dependence unit for intensive monitoring and care and review of skills and experience of hospital anaesthetists to ensure that operating theatres coverage is always with competent and experienced people to prevent anaesthetic deaths. Maternal deaths review forms are also reviewed regularly after initial reviews to ensure that they are filled appropriately before they are submitted to appropriate levels of the health system.

The finding of incompletely filled review forms did not provide enough information to understand all circumstances surrounding the deaths as lessons gained would not lead to better understanding of the management provided to women and identification of dysfunctions to address in order to avoid similar deaths in future in the absence of case notes from the deceased patients’ files. Two main reasons were identified to explain this finding. In some cases, important information was not recorded at the referring health facility and the situation did not allow obtaining such details after the woman had died (an indication of poor referral details); but in some, extraction of the information from the patient records into the review forms was incomplete. Unfortunately, regular review of all filled forms lacked thus not allowing identification of such weaknesses to recommend appropriate and timely remedies. Although measures to avoid missing information on various actions decided during the reviews and how they are followed-up when they are filled has been addressed at Bugando Medical Centre, similar weaknesses likely occur in other facilities in the country. We recommend that the on-going-review of the national maternal and perinatal deaths review guidelines recognize this as a weakness and include measures to avoid it. Also quarterly, semi-annualy or annually reports may involve few cases for an in-depth analysis and planned analysis after some years may be a more pragmatic option to consider, especially is hospitals with limited caseloads.

The finding that approximately 80 % of all deaths occurred when the deceased had stayed in the hospital for at least a day and almost a third stayed for at least four days highlights the quality of care women receive at this hospital and in other facilities referring such women. Measures to improve the quality of care at health facilities in the lake zone are imperative for better maternal survival. The on-going Lake Zone Maternal Mortality Reduction Initiative by Bugando Medical Centre with over 35 district and regional hospitals aiming at improving the quality of care in these health facilities is a welcome measure. Results from a one year experience of implementing this intervention are encouraging and will be reported separately. Additionally, country level analysis of the coverage, equity and quality of key reproductive, maternal, newborn and under-five children indicators shows that the lake and western zones are the least performing in the country impacting negatively on the overall country MDG4&5 progress. [19] Subsequently, pertinent issues contributing to the sub-optimal progress are being addressed, including but not limited to ensuring availability of essential medicines, equipment and other supplies; recruiting, deploying and retaining of skilled providers; improving community engagement in service availability and utilization and improving basic infrastructure for effective referral through increased regional and council health budgets and the Big Result Now Initiative. The latter is a special presidential initiative to accelerate progress in key national sectors, including health.

Of equal note are the relatively low documented AIDS-related deaths in this hospital. Recent estimate suggest that such deaths may account for almost 6 % of all deaths in the country. [20] Effective PMTCT services in this part of the country may partly explain this although errors in ascertaining causes of deaths cannot be ruled out. However, similar to previous studies in the country, direct causes of deaths accounted for most deaths [1, 3].

The finding that late referral of patients from health facilities occurred in almost a third of all deaths indicates that improving the quality of care in the referring facilities is imperative for better maternal survival in this setting. Training of available staffs in these facilities for essential competences in management of pregnancy-related complications and quick referral where required is a short term option but good supportive supervision is vital to reinforce the acquired skills and to identify and address challenges in appropriate skills and within the health system. Ensuring that health facilities have the necessary supplies and equipment for managing maternal complications is another necessary intervention.

The main strength of this assessment is to highlight the causes of maternal deaths and related factors which informed subsequent steps to improve on the management of women with obstetric and related complications in the hospital. As a result, periodic analysis of the filled maternal and perinatal deaths review forms has been included in the hospital quality of care assessment and other measures instituted as required by the current national maternal and perinatal death review guidelines and plan for accelerated reduction of maternal, newborn and child deaths [13, 21].

Some limitations of this study are worth mentioning. Collection and analysis of retrospective data posed some challenges. Notably, the quality of data could not be controlled in retrospect but very much depended on the previous practice and mechanism of ensuring good quality data. Additionally, like what has have been reported elsewhere in Africa [22–25], we encountered incompletely filled MDR forms, errors we had no control over. The forms were also of different review cycles and possibly done at different times of staff competences and as a result, consistency in case management might have lacked. Routine facility MDRs at Bugando Hospital and indeed in the entire lake zone and countrywide do not involve collecting in-depth community information on factors associated with maternal deaths but highlights of what might have happened before the woman reached the health facility. Subsequently, available data may not allow understanding of factors that might have prevailed before contact with the health system thus unable to comprehensively address challenges women face in accessing available services.

## Conclusions

We conclude that maternal deaths reviews and documentation of information from such reviews at Bugando Medical Centre in the period 2008–2012 were not always comprehensive enough to provide all the necessary information required in the current national guidelines and to understand the quality of care women received before demise. This is currently addressed through improving providers understanding of the essence of the reviews, the review process as well as documentation of information from the reviews, including proposed actions to prevent similar deaths in future. The roll-out of the new national guidelines should also aim to build strong capacity for tertiary institutions like Bugando, so that they function as resource centres for training skilled professionals in maternal and perinatal death reviews.
